# Molecular and Structural Characterization of an Immunopurified Telomerase from *Leishmania major* and the Effect of Telomerase Inhibitors

**DOI:** 10.3390/microorganisms13020357

**Published:** 2025-02-07

**Authors:** Riward Campelo Morillo, Liliana Casique, Katherine Figarella, José Luis Ramírez

**Affiliations:** 1 Institute of Advanced Studies, Ministerio del Poder Popular para Ciencia y Tecnología (MINCYT), Caracas 1010, Venezuela; riwardc@gmail.com (R.C.M.); lcasique@usb.ve (L.C.); kfigarella@gmail.com (K.F.); 2 Departamento de Biología, Universidad Simón Bolívar, Caracas 1080, Venezuela; 3 Instituto de Biología Experimental, Universidad Central de Venezuela (UCV), Caracas 1040, Venezuela

**Keywords:** *Leishmania major*, protozoan parasite, telomerase, heat resistant, special structure, activity inhibitors

## Abstract

*Leishmania major* is the etiological agent of cutaneous leishmaniasis (CL) in several countries in Asia and Northern Africa. The disease is considered a zoonotic infection where rodents are the reservoirs and phlebotomine sandflies are the vectors. Once inside the human body, the parasite multiplies inside the macrophages of infected patients, but the disease eventually cures spontaneously, leaving scars where the phlebotomine bites occurred. Given the importance of the replicative forms in the parasite’s cell cycle, here, we decided to study the enzyme telomerase, which has the critical role of replenishing the chromosomal telomeric ends during cell replication. To this aim, we first conducted partial purification using Sephacryl-300 HR gel filtration, which allowed us to determine that the telomerase activity eluted as a 600 KDa complex. Second, we characterized an immunopurified *L. major* telomerase, and to try to explain some of our findings, we performed modeling studies using Alfa fold 3, Pyre2, and Swiss Protein Model. Finally, considering the similarity between the catalytic site of *Leishmania* and *Homo sapiens* telomerase, we decided to test typical inhibitors of human telomerase on the purified enzyme and promastigote cell forms, confirming that MST-312 and TMPYP4 efficiently inhibited *L. major* activity and arrested cell growth in *Leishmania* promastigotes. Our findings confirm the importance of telomerase activity in *L. major*’s replicative forms and suggest the possibility of using drugs previously tested on human telomerase to treat CL.

## 1. Introduction

In humans, cutaneous leishmaniasis is caused by infection with the parasite *Leishmania major*. The disease is considered a zoonotic infection whose reservoirs are rodents living in the arid savannahs of Northern Africa and Asia, and the transmission to humans occurs via the bite of female phlebotomine sandflies [1]. Although cutaneous leishmaniasis caused by *L. major* infections often heals spontaneously, it leaves disfiguring scars on patients’ faces and bodies, and clinical complications can arise in immunocompromised or undernourished patients [2]. In most eukaryotes cells, after each replication, reverse transcriptase telomerase restores the chromosomal ends (telomeres). The basic telomerase holoenzyme is composed of the reverse transcriptase sub-unit (TERT: telomerase reverse transcriptase) and RNA sub-unit (TER: telomerase RNA), which serves as a template for the addition of six nucleotide repeats at the 3′ telomeric ends [3]. Metazoan somatic cells are programmed such that the number of mitotic divisions is proportional to the number of telomeric repeats [4]; however, cancer cells avoid this mitotic clock [5] either by overexpression of telomerase [6] or by using alternative strategies such as telomere replacement by homologous recombination [7,8]. In the case of unicellular organisms like *L. major*, the population survival of replicative forms depends on continuous cell divisions; thus, telomerase expression should always be active. However, given the peculiar structure of *Leishmania* telomeres [9,10] and the low levels of telomerase activity and processivity registered in previous works for this parasite [11], there is a possibility that the parasite can use alternative mechanisms for telomeric recovery [9] (Figure S1). If telomerase activity is essential for *L. major*, it could provide health practitioners with an important chemotherapeutic target.

In our previous works with nuclear extracts [12], we showed that by adjusting some assay parameters, including the dilution of nuclear extracts due to the presence of unknown inhibitors, *L. major* telomerase was highly active, reaching levels comparable to those observed in human cancer cells. Here, we used immunoprecipitation techniques to purify and characterize a highly active *L. major* telomerase, and to try to explain some of the physicochemical characteristics of this enzyme, we carried out structural modeling studies. Finally, we confirmed that typical inhibitors of human telomerase blocked the purified *L. major* telomerase activity and arrested the cell growth of promastigote forms.

## 2. Materials and Methods

### 2.1. Parasitic Cells

*L*. *major* strain Friedlin (MHOM/JL/80/Friedlin) was kindly donated by Dr. Ángela Cruz, University of Sao Paulo, Riberao Preto, Brazil. Cells were harvested in log phase at 25 °C in RPMI-1640 medium (Gibco-BRL) supplemented with 10% heat-inactivated fetal calf serum (Invitrogen, Thermofisher, Waltham, MA, USA).

### 2.2. Preparation of Nuclear Extracts from L. major Promastigotes

*L. major* nuclear extracts were prepared as previously described [13], with modifications [12].

### 2.3. Preparation of Fractions Enriched in L. major Telomerase Using Sephacryl-300HR Gel Filtration

Based on *L. major* telomerase thermostability, we incubated nuclear extracts at 70 °C for 15 min, followed by centrifugation at 15,000 rpm for 15 min to precipitate denatured proteins. Next, we loaded 300 µL (0.5 mg of protein/mL) of the supernatant on a pre-calibrated Sephacryl-300HR (Merck Life Sciences, Darmstadt, DE) column and filtered the sample at an entry flux of 0.2 mL/min and exit flux of 0.15 mL/min.

### 2.4. Production of Anti-Telomerase Antibodies

To produce antibodies against the TERT subunit of *L. major*, we cloned a fragment corresponding to the first 900 bp of the specific N-terminal portion of telomerase using the gene sequence information deposited in GenBank (GenBankTM XM_001686924) [14]. The recombinant plasmid was transferred into *E. coli* BL21(DE3) pLysS cells (Invitrogen, (Invitrogen, Thermofisher, Waltham, MA, USA), and the cloned protein fragment was over-expressed following the protocols of Sambrook and Russell (2001) [15]. The over-expressed TEL-1 peptide was fused to a glutathione synthetase (GST) enzyme sequence to facilitate its purification through Bulk and RediPack GST purification modules (GE Health Care, Pistcataway, NJ, USA). To obtain polyclonal antibodies, the TERT fragment was inoculated into rabbits following standard procedures. After 60 days, blood was drawn via cardiac puncture, and the antiserum was separated. The antibodies were purified by immunoabsorption and used in immunoprecipitation and immunodetection experiments.

### 2.5. Immunoprecipitation Assays

For immunoprecipitation and immunodetection assays, we incubated Protein-A Sepharose^TM^ beads (VWR, Radnor, PA, USA) with the anti-TEL1 antibody or preimmunize serum at a ratio of 5:1 for 1 h at room temperature. Next, 250 µg of nuclear extract (preheated at 65 °C or not for 5 min) was incubated with 12 µL of immunized rabbit serum; then, we added 60 µL of 50% p/v of Sepharose-protein A beads previously treated with the preimmunized serum for 16 h at 4 °C with shaking. The beads were centrifuged at 1000× *g* for 30 s and then washed 20 times with 10 mM Tris-HCl pH 7.5, 1 mM MgCl_2_, 1 mM EGTA, 0.2% *v*/*v* Nonidet NP-40, 40 mM NaCl, 10% Glycerol, and protease inhibitor cocktail (Sigma, St. Louis, MO, USA). Finally, the beads were suspended in 1 X TMG buffer (10 mM Tris-HCl, pH 7.5; 1 mM MgCl_2_; Glycerol 40%) at a 100:1 ratio.

**Figure 1:**
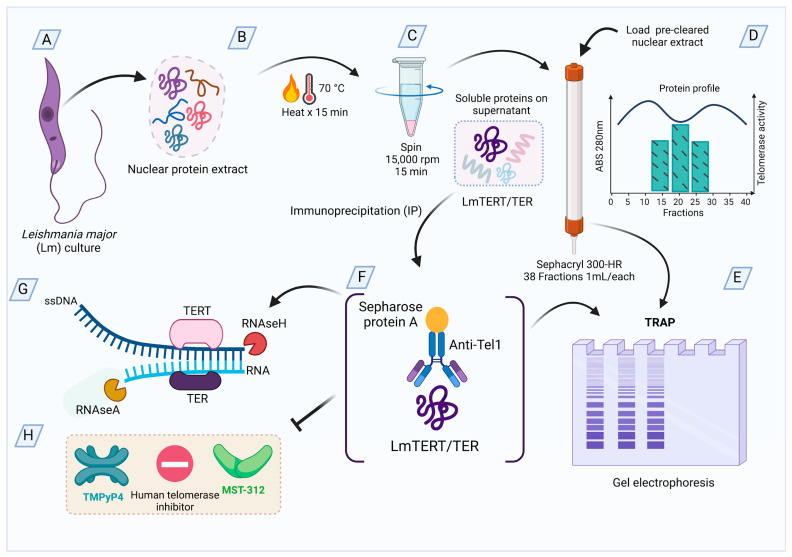
*L. major* telomerase purification procedures. (**A**) Nuclear protein extracts were prepared from *L. major* cultures. (**B**) The nuclear extracts were heated at 70 °C for 15 min (**C**). The extracts were pre-cleared by centrifugation at 15,000 rpm for 15 min. (**D**) The soluble protein fraction was loaded onto a Sephacryl 300-HR column and separated by chromatography. (**E**) Eluted fractions were tested for telomerase activity. Alternatively, pre-cleared nuclear extracts were subjected to immunoprecipitation (**F**) using anti-Tel1 antibodies coupled to Sepharose Protein-A pearls. (**G**) The effects of RNase A and RNase H on immunopurified telomerase activity were tested. (**H**) An *in vitro* assay was conducted to evaluate the effect of human telomerase inhibitors on telomerase activity.

A summary of the purification procedures is shown in Figure 1.

### 2.6. Electrophoresis and Western Blots of L. major Telomerase

For Western blotting experiments, 20 µL of precipitated beads was treated with 1% Triton X-100, resuspended in 8% SDS buffer, and ran in 10% polyacrylamide gels without further denaturation procedures. The bands were transferred to nitrocellulose membranes according to Sambrook and Russell (2001) [15]. Membranes were blocked with 5% low-fat dry milk in Tris-buffered saline (TBS) overnight at 4 °C. After blocking, the membranes were incubated with a 1:500 dilution of primary antibody (anti-TEL1) for 1 h at room temperature, then washed three more times for 15 min with TBS-Tween and incubated for 1 h with anti-rabbit antibodies labeled with horseradish peroxidase in a 1:5000 dilution (Santa Cruz Biotechnology, Dallas, TX). After this incubation, we washed the membranes three times for 15 min with TBS-Tween (Tris 200 mM, 420 mM NaCl) and then developed in the dark with SuperSignal^®^ West Pico Chemiluminescent Substrate, Pierce, Thermofisher Waltham, MA, USA).

### 2.7. Telomerase Detection Assay

We used two assays: the first was the TRAPeze XL^®^ kit (Sigma, St. Louis, MO, USA ) following the manufacturer’s instructions. This kit uses a protocol modified from the original TRAP (Telomeric Repeat Amplification Protocol) [16], in which the primer reaction was supplemented with the oligonucleotide 5′-ATCCGTCGAGCAGAGTTAGGGTTAGGG-3 [14], while the reverse primer consists of telomeric sequences bearing an Amplifluor^®^-conjugated (a hairpin structure is formed in this primer between its 3′ end between the fluorescein fluorophore and the DABSYL (4-(dimethylamine)azobenzene sulfonic acid) quencher. To analyze the activity of *Leishmania* telomerase, we used 2 µL of beads with immunoprecipitated telomerase [17]. The reaction mixture was incubated at 30 °C for 30 min. The amplification of telomerase products was performed using 2 units of Taq polymerase (Invitrogen) and a thermocycling program consisting of 36 cycles of 94 °C for 30 s, 55 °C 30 s, 72 °C 1 min. The telomerase PCR products were resolved by capillary electrophoresis using Performance Optimized Polymer 4 (POP-4™) (Applied Biosystems, Waltham, MA, USA), and the total fluorescence emission was detected on an ABI PRISM™ 310 automated genetic analyzer (Applied Biosystems, Waltham, MA, USA). Total activity was calculated by summing the areas of the peaks showing the typical 6 bp incremental ladder of telomerase activity. The size of the fragments was calculated using the LIZ™ Size Standard (Applied Biosystems). As a positive control for telomerase activity, we used nuclear extracts of PC3 human cancer cells, prepared following the directions for CHAPS TRAPeze XL^®^ (Chemicon International).

The second assay used the same PCR conditions for the Ts extension primer ending in 5′TTAGGG-3′ and the non-labeled reverse primer 5′CCCTAACCCTAACCCTAA-OH-3′. The PCR products were resolved in 12% polyacrylamide gels and stained with Sybr-green (Sigma) in a ratio of 1:10,000, and the detection and band area calculations were conducted with a Typhoon 9410 High-Performance Gel and Blot Imager (GE Health Care, Pistcataway, NJ, USA).

### 2.8. RNase H Assays

For the RNase H assay, we used the TSR8 primer, which is similar to the TS primer but has 7 hexameric repeats at the 3′end, and a sequence complementary to the 9 nucleotides of TER’s anchoring sequence (underlined). AATCCGTCGAGCAGAGTTATTAGGGTTAGGATTAGGTT(TTAGGG)7 [10]. The immunopurified enzyme was first heated to 65 °C, followed by incubation with 2 attomoles of the primer TSR8 at 28 °C for 30 min. Finally, we added increasing RNase H units and incubated at 37 °C for 30 min.

### 2.9. Structural Simulations

The amino acid sequence of *L. major*’s TERT protein was obtained from UniProtKB accession IDs QNN2R7(1053aa) and Q4Q122 (1451 aa). In a pBLASTp alignment against the PDB database, we found three *Homo sapiens* TERT structures with the lowest E-scores (PDB ID 7bga, 7trd, and 7qxa), followed by the *Candida tropicalis* telomerase (PDB ID 6zd2). The aligned region of *L. major*’s TERT included the reverse transcriptase domain (400 aa) with a 26% sequence identity to the human protein. Homology modeling of this region using the Swiss-Model server [18] revealed that the human protein structures PDB ID 7qxa and 7trd served as the best templates to model *L. major*’s Q2NNR7 and Q4Q122. Additionally, using the AlphaFold3 server [19], we obtained a non-template-based structure for residues 434 to 1410 of Q4Q122. We performed a Ramachandran plot analysis (PROCHECKv.3.5.4) of the 3D models obtained, including the AlphaFold-3 structure ID AF-Q2NNR7-F1. For the final quality assessment of the models, we used SAVES v6.0 (https://saves.mbi.ucla.edu/ accessed on 11 November 2024) and Prosa-web (Table S1). For an overall 3D model validation, we used QMEAN 4.3.1 and MolProbity 4.5.2 [20]. Further refinement of the model was performed as suggested by Fiser (2010) [21] and Haddad et al. (2020) [22]. Next, to refine the location of RT-TERT motifs in the parasite protein, we aligned our 3D model, and the TERT proteins PDB ID: 7qxa, 3du5, 6zd2, 6d6v using PROMALS3D, DALI server, and RCSB structural pairwise alignment tool. Given the high tridimensional conservation in the finger and palm domains with the type II intron from *Geobacillus stearothermophilus* (PDB ID 7k9y) [23], the transcriptase reverse of this organism was included in the analysis.

### 2.10. Assays with Telomerase Inhibitors

Human telomerase inhibitors TMPYP4 (5,10,15,20-Tetrakis(1-methylpyridinium-4-yl) porphyrin tetra(p-toluenesulfonate) (AbcamBiochemicals, Cambridge, UK) [24] and MST-312 (N-[3-[(2,3-dihydroxy phenyl)-oxomethyl]amino]phenyl]-2,3-dihydroxy benzamide (Calbiochem, Gillingham, UK) [25] were added to the purified *L. major* telomerase (pre-heated at 65 °C for 5 min) at concentrations ranging from 0.1 to 4 µM and incubated at 25 °C for 30 min. The telomerase assays were performed as previously described.

### 2.11. Effect of Telomerase Inhibitors on Promastigotes of L. major

To evaluate the effect of the telomerase MST-312 inhibitor on cell proliferation, we cultivated *L. major* promastigote cells in the presence of different concentrations of the inhibitor for seven weeks. MST-312 inhibitor was reconstituted in DMSO, keeping the final DMSO concentration below 1%. The same concentration of DMSO was added to the cells used as a control. Cultures were started with 2 × 10^6^ cells per ml. The cell density of each culture was controlled weekly by counting the parasites in a hemocytometer, and the percentage of inhibition versus the control cells was determined. At each point, the cultures were diluted with fresh medium, and the drug was renewed. In the fourth week, we doubled the drug concentration [25].

## 3. Results

### 3.1. Sephacryl-300HR Gel Filtration of L. major Nuclear Extracts

**Figure 2:**
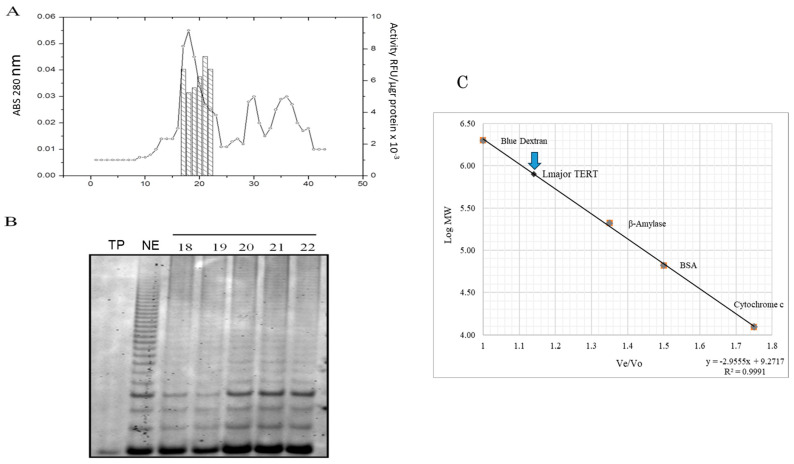
Partial purification of *L. major* telomerase from a nuclear extract heated at 65 °C using Sephacryl S-300 HR chromatography. (**A**) The 280 nm absorbance profile and telomerase activity (in bars). (**B**) Sybr-Green-stained 12% polyacrylamide gel showing the products of telomerase activity in fractions 18 to 22. The first lane shows the activity in preheated (TP) total cell proteins: second lane (NE) nuclear extract preheated at 65 °C. (**C**) Log-Plot to calculate the approximate molecular mass of *L. major* telomerase complex (pointed with blue arrow).

Figure 2A shows the 280 nm absorbance and telomerase activity profiles from Sephacryl-HR300 chromatography of the supernatant of an *L. major* nuclear extract after pre-heating and centrifugation (Section 2.3). We collected 38 1 mL fractions and assayed them for telomerase activity. The peak of telomerase activity (Figure 2B) eluted with a molecular mass close to 600 kDa (blue arrow Figure 2C). When we pooled the fractions with the highest activity and performed a Western blot assay, we detected many contaminant proteins and a weak telomerase band (Figure S2).

### 3.2. Immunopurified L. major Telomerase

**Figure 3:**
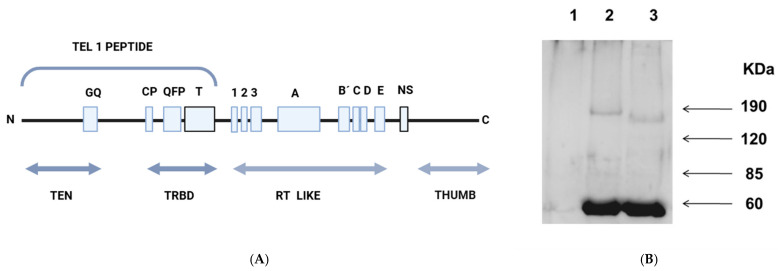
Schematic representation of *Leishmania major* TERT gene. (**A**) The bracketed segment TEL-1 corresponds to the *Leishmania major* telomerase peptide used to obtain anti-TERT antibodies. TEN, Telomerase N-terminal; TRBD, Telomerase RNA binding region; RT like, reverse transcriptase domain, and the thumb closing the catalytic site. (**B**) Western blot of 10% polyacrylamide gel with the proteins eluted from the Sepharose-protein-A pearls after treatment with 1% *v*/*v* Triton^®^ X-100. Lanes are as follows: 1, proteins retained by Sepharose-A pearls without anti-telomerase antibody after incubation with a nuclear extract; 2, proteins eluted after mixing the nuclear extract with the pearls coupled to anti-Tel-1 antibody and heated at 65 °C; 3, similar to 2, but the sample was kept at room temperature. Samples were loaded with loading buffer (50 mM Tris-HCl pH 6.8; 2% *w*/*v* SDS; 8% *v*/*v* Glycerol) without further denaturing procedures.

Since the Sephacryl filtration experiments rendered low yields and had many contaminant proteins, we opted for immunoprecipitation techniques to isolate *L. major* telomerase (see Section 2.5). The immunopurified enzyme recovered from Sepharose-A pearls, pre-warmed (or not) at 65 °C, was resuspended in SDS buffer and ran in SDS-PAGE gels. Figure 3B (lanes 2 and 3) shows a clear protein band of M*r* 180,000; as occurs with crude extracts, the unheated sample migrated as a band with a lower M*r* of 160,000. Although both values were close to the calculated molecular mass of 159,000, (−/+ 10%), the heated enzyme consistently exhibited a retarded migration. The sample treated with preimmunized serum is shown in Figure 3 lane1. The strong band around 60 kDa shown in lanes 2 and 3 corresponds to the antibodies’ heavy chain. The changes in activity and electrophoretic mobility are reversible because the protein recovered the characteristics of the non-heated enzyme once cooled.

### 3.3. Measurement of Telomerase Activity in Immunopurified Enzyme and the Effect of the Extension Primer Sequence

In cloning the telomeres of *L. donovani* [10] and *L. major* [26], we successfully used a telomeric adaptor complementing the sequence 5′-GGTTAGGGT-3′. However, when we tested *L. major* telomerase activity using an extension primer ending with this sequence [12], we failed to produce the typical 6 bp incremental ladder of telomerase activity. The Ts primer 5′-AATCCGTCGAGCAGAGTT-3′ included in the original Kim’s TRAP assay also gave weak signals, while the best results were obtained with the extension primer 5-AATCCGTCGAGCAGAGGG-3′ (Figure S3).

### 3.4. Effect of Temperature on L. major Immunopurified-Telomerase Activity

**Figure 4:**
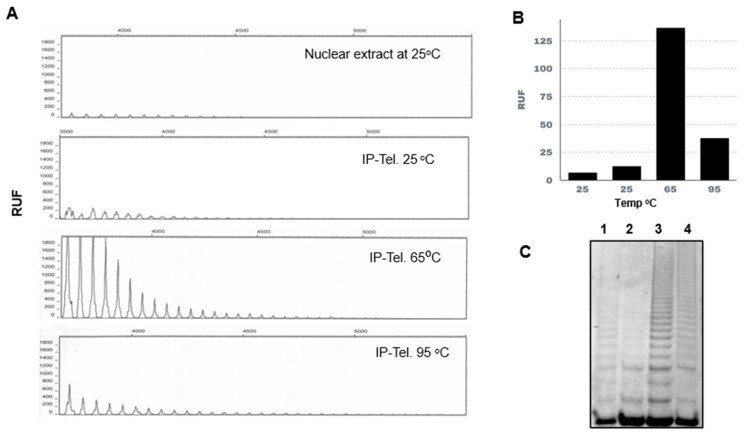
Effect of temperature on *L. major* telomerase activity. (**A**) Capillary electrophoresis of the products of the immune-precipitated telomerase (IP-telomerase) activity. The first panel shows the activity in the nuclear extract at 25 °C. The rest of the panels show the activity of the IP enzyme at different temperatures. (**B**) Bar graph summing the areas of the relative units of fluorescence (RUF) peaks in A. (**C**) Similar to the experiment in A, but the products were resolved in a 12% polyacrylamide gel stained with Sybr-Green. Lanes: 1. nuclear extract at 25 °C; 2. IP-telomerase at 25 °C; 3. IP-telomerase heated at 65 °C; 4. IP-telomerase heated at 95 °C.

We tested the telomerase activity of the *L. major* immunopurified enzyme at different temperatures. As shown in Figure 4, the nuclear extract A (7 RFU) and the purified enzyme (Figure 4B) second panel (13 RFU) have low activity at 25 °C, but once heated at 65 °C, the activity increased by more than 100-fold (third panel) (130 RFU). The enzyme pre-heated at 95 °C for 30 min retained some activity (fourth panel) (28 RFU). A duplicate of this experiment was performed using the classical TRAP, and products were visualized using polyacrylamide gels, as shown in Figure 4C.

### 3.5. RNases A and H Treatment

**Figure 5:**
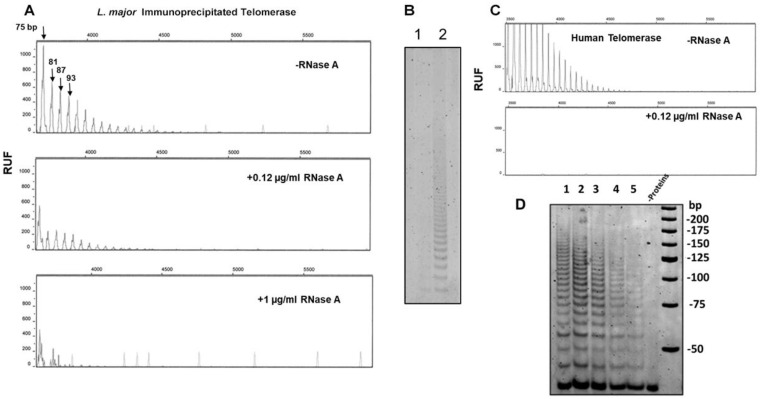
Effect of RNases-A and H on *L. major* IP-telomerase activity preheated at 65 °C. (**A**) Capillary electrophoresis of *L. major* IP-telomerase after treatment with RNase-A: 1, no RNase-A; 2, 0.12 ug of RNase-A: 3, 1 µg RNase-A; (**B**) 12% polyacrylamide gel electrophoresis of IP-telomerase activity: 1. Non-heated IP-telomerase treated with RNase-A 0.12 ug/mL, 2. IP-telomerase previously heated at 65 °C and treated with RNase-A 0.12 ug/mL; (**C**) effect of 0.12 ug/mL of RNase-A on the telomerase activity of human PC3 cancer cells; (**D**) effect of RNase H on the IP-telomerase. The 12% *v*/*v* polyacrylamide gel showing the products of the activity of the IP-telomerase after increasing concentrations of RNase-H. IP-telomerase was previously heated at 65 °C for 10 min and incubated with 2 attomoles of oligonucleotide TSR8. The first lane is the control experiment without RNase H (first lane); lanes 2 to 5 show activity in the presence of increasing (0.5, 1, 2 and 4 RNase H units) concentrations of RNase H; in lane 6, no protein was included in the reaction.

When the purified *L. major* telomerase was heated to 65 °C and incubated with 12 µg/mL of RNase A (Figure 5A), we observed a decrease in activity, but to achieve complete inhibition, we added 0.1 mg/mL of RNase A. Figure 5B shows a polyacrylamide gel with the products of the immunopurified enzyme treated as before (1, non-heated enzyme with 12 µg/mL of RNase A, and 2, heated at 65 °C and the same enzyme concentration). In the positive control consisting of a cell lysate of human prostate cancer cells (PC3), the addition of 12 µg/mL of RNase A eliminated the activity (Figure 5C).

RNase H acts on RNA-DNA hybrids, and its action in *L. major* telomerase should be on the TER subunit annealed with the telomeric substrate. As shown in Figure 5D, a clear reduction in activity was only observed when four units of this enzyme were added. Again, this assay revealed restricted access to RNases when the enzyme was heat-activated.

### 3.6. Structural Simulations to Obtain the Structural Models of L. major

**Figure 6:**
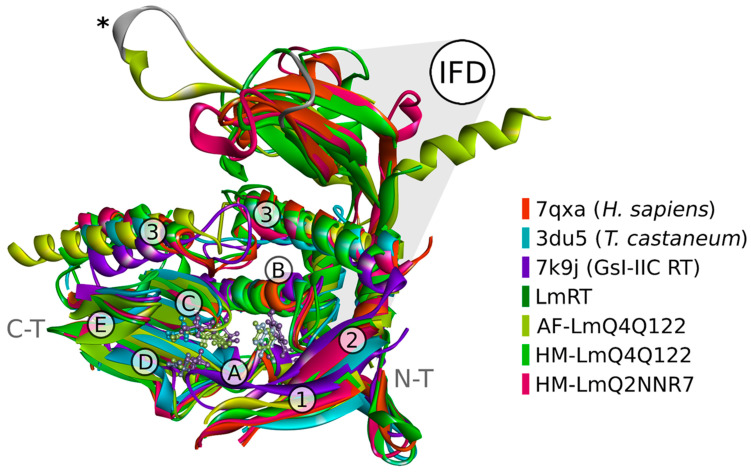
The structures obtained from UniProt databases Q2NNR7 and Q4Q122 (magenta and green ribbons, respectively) are superimposed over the human RT (PDB ID: 7qxa), light orange, and *Tribolium castaneum* (PDB ID: 3du5), blue. The homology models HM-LmQ2NNR7 and HM-Q4Q122 for the RT domain of *L. major* TERT were inferred by SWISS-MODEL using the human RT (PDB ID: 7qxa and 7trd) as a template. The more extensive model for the TERT protein (AF-Q4Q122) was developed with AlphaFold-3. The *L. major*’s RT structure, reported by Da Silva et al. (2023) [30], was included using the pairwise alignment tool as indicated in the Section 2.9. Also, the group II intron-encoded non-LTR-retroelement reverse transcriptase from *Geobacillus stearothermophilus* (PDB ID: 7k9y, purple) is overlaid with the RT-TERT structures. The right panel shows the color scheme used to visualize each backbone in ribbon representation of the RT domain; numbers (1–3) and letters (A–E) indicate each motif. IFD: insertion finger domain (grey outline); Domains A and C show catalytic (3 Asp) and gate (Y) residues in ball and stick representation, denoting the conserved spatial arrangement of these residues among the different TERT proteins. An asterisk (*) indicates the location of the Cys-Arg-Gly-Gly-Cys motif. Images were generated using the Discovery Studio Visualizer version 24.1.0.23298. Structures in PDB format can be obtained upon request.

From the structural analysis, we selected structures that were superimposed with BIOVIA Discovery Studio software, version 24.1.0.320629. We omitted some disordered (non-structured) regions of *L. major* TERT to facilitate tertiary structure visualization (Figure 6). The structural analysis of *L. major* TERT sequences showed that this protein’s finger and palm domain had a high structure similarity to the analog domains from the *Homo sapiens* protein. Still, the RT-ring that accommodates the DNA-RNA duplex seems to have a closer conformation. According to this theoretical model, *L. major* protein might have tighter binding of the TER sub-unit after heat treatment, exposing hydrophobic or positive charged regions that might affect its migration on SDS-PAGE; a more compact structure might also be responsible for the restricted access to RNases A and H. In the structural analysis, we found a significant difference in the interconnecting loop between *Homo sapiens* and *Leishmania* beta sheets of the IFD domain (Figure S4). This unstructured loop region has high sequence and length variability across species [27]. In addition, this loop is larger in *L. major*, has a different spatial orientation, and has a Cys-Arg-Gly-Gly-Cys motif from position 974 to 978 (reference sequence Q4Q122). The MOTIF search tool from the GenomeNet web server indicated that this sequence is present in enzymes that can sense the redox state of the cell (UniProtKB O19132) [28], or in proteins (UniProtKB Q5Z5Q3, O94813) or nucleic acid (UniProtKB ID P9WJ78, Q03274) binding motifs. A closer inspection of the catalytic sites (Figure S5) revealed that despite having high amino acid sequence conservation, *L. major*’s cysteine residue C935 substitutes alanine A716 of the *Homo sapiens* protein, which, in turn, precedes the tyrosine residue Y936 (Y717 in *Homo sapiens* protein). *Tribolium castaneum* telomerase [29] Y936 is considered a “gate” for selecting deoxynucleotides over ribonucleotides. Cysteine C935 substitution was also present in the TERT sequences of *Tetrahymena thermophila*, *Candida albicans*, and *T. brucei*. Oxidized cysteines play multiple roles in protein function regulation that are generated by changes in the redox state of the environment [28]; ascertaining the relevance of these observations will require significant additional work.

### 3.7. Telomerase Inhibitors of L. major Telomerase Activity

**Figure 7:**
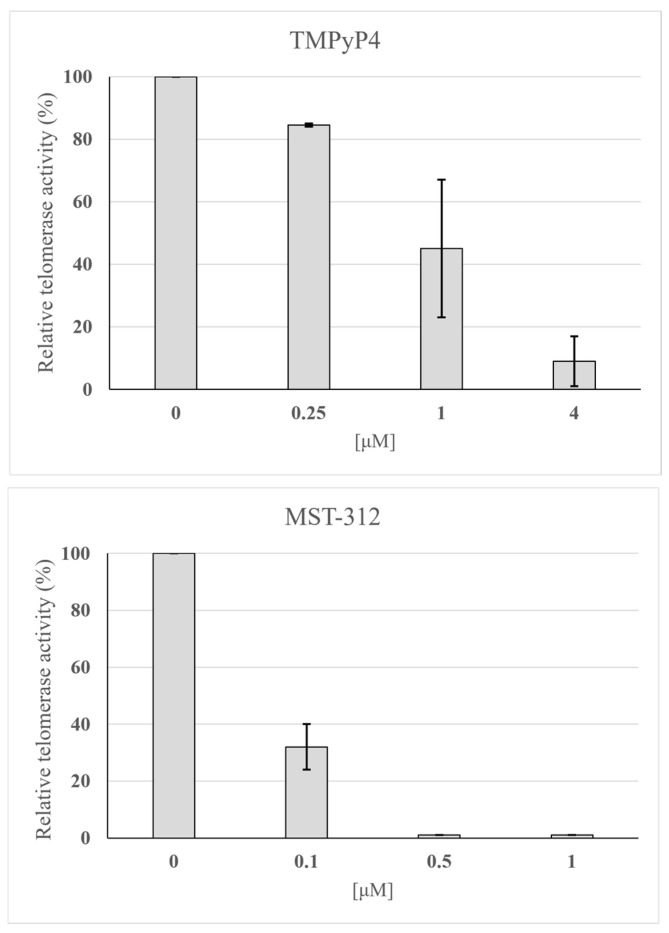
Effect of human telomerase inhibitors MST-312 and TMPyP4 on *L. major* IP-Telomerase. The means and ±SEM of two replicas is shown.

Next, we sought to determine whether the human telomerase inhibitors TMPyP4 [24] and MST-312 [25] could inhibit *L. major* telomerase activity. As shown in Figure 7, both compounds effectively abrogated *L. major* telomerase activity. However, MST-312 proved to be more potent than TMPyP4, achieving complete inhibition of telomerase activity at 0.5 μM—eight times lower than the concentration required for TMPyP4. Based on these results, we selected MST-312 for further experiments.

### 3.8. Effect MST-312 on L. major Promastigote Cell Growth

**Figure 8:**
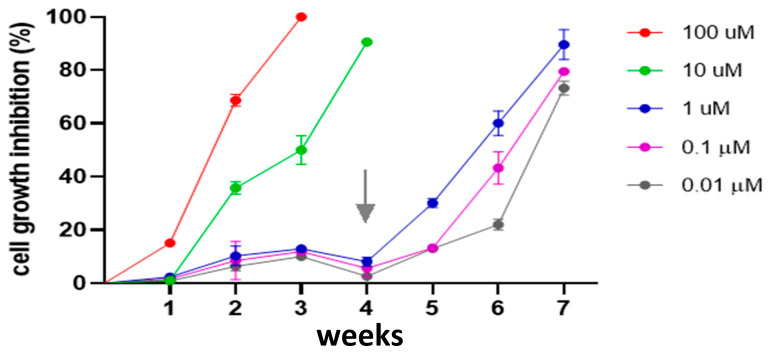
Effect of human telomerase inhibitor MST-312 on cell growth of *L. major* promastigote forms. The arrow points to the week at which the culture medium was refreshed, and the inhibitor concentration was doubled.

To test the effect of telomerase inhibitors on *L. major* promastigote cell growth, we used inhibitor MST-312. First, we treated *L. major* promastigote cells with decreasing concentrations (100, 10, 1, 0.1, and 0.01 µM) of MST-312. In the experiment shown in Figure 8, we only observed significant growth inhibition during the first three weeks with the highest inhibitor concentrations (100 and 10 µM). Considering the experience of Seimiya et al. [25] with human cancer cells, starting from the fourth week, we doubled the inhibitor concentration, obtaining 70% growth inhibition at 0.02 µM (20 nM).

## 4. Discussion

Telomerases are not very abundant molecules; even in cancer cells such as HEK 293T (256 telomeres) and HeLa (304–320 telomeres), their approximate number is 240 molecules per cell, i.e., there are fewer telomerase molecules than the number of telomeres [31]. A similar finding was reported for yeast [32] and, as suggested by [31], a balance is reached during the S phase of the cell cycle. In the case of *L. major* multiplicative phases, we expect as a minimum an active telomerase at each of the 144 telomere ends [33], a fact that makes telomerase purification a difficult task.

In an attempt to purify and assess whether *L. major* telomerase existed as a monomer or a protein complex, we performed Sephacryl-300HR gel filtration chromatography on a preheated (at 70 °C) nuclear extract, revealing that the telomerase activity eluted as a complex with an approximated molecular mass of ~600 KDa (Figure 2). The size of this complex can be explained by its association with other proteins and a large TER subunit. Nonetheless, the reported size for the presumptive *L. major* TER is 2100 nucleotides [34], which would put this complex above the estimated value. Similar results were reported for *Trypanosoma cruzi* and *L. amazonensis* [35,36]. Preliminary mass spectrometry of the enriched telomerase fractions identified several candidates interacting proteins, suggesting that the telomerase complex in *L. major* may include associated factors. Further investigation is needed to confirm these interactions. In conclusion, this purification procedure has low yields, and the product has many contaminant proteins.

Telomerase activity has been analyzed in nuclear extracts from *L. tarentolae*, *L. major*, *Trypanosome brucei* [11], *L. amazonensis* [36], and *T. cruzi* [35]. Although *T. brucei*’s activity was robust, *Leishmania* [11,36] and *T. cruzi* [29] exhibited low processivity. These are counterintuitive findings given that the replicative forms of these parasites divide very actively; in addition, from a sequence analysis and structural simulations of the catalytic site of *T. brucei*, *T. cruzi*, and *Leishmania*, [30], there are no important changes in key catalytic aa residues (Figure S5) that may explain the low activities and processivity registered for the last two parasites. There are some differences; i.e., in *L major*’s motif 3, there is a change of an R for Q in position 867. However, major changes in the TEN domains have been observed; da Silva and colleagues [30] speculate that this region might be responsible for the processivity differences among telomerases.

In our previous works, in control experiments aiming to heat-inactivate telomerase activity in nuclear extracts of *L. major* and *T. cruzi*, we obtained the opposite result, i.e., the activity increased in a range from 25 °C to 55 °C, peaking at 65 °C [11,37]. Thus, we optimized the TRAP reactions, adjusting the following parameters: first, we diluted the nuclear extracts, used an extension primer ending in -GGG-OH-3′, and preheated the extracts at 65 °C for 5 min. Under those conditions, the telomerase of *L. major* reached activity levels comparable to those of PC3 cancer cells. The purified *L. major* telomerase using a specific polyclonal antibody against the TEN region of TERT (Figure 3) resulted in a specific and efficient procedure that allowed us to confirm the thermal stability and heat activation of this enzyme (Figure 4) and its resiliency to be inactivated by RNases treatments (Figure 5).

Our assays with different extension primers revealed an interesting fact; i.e., despite variable telomeric terminus in *L. donovani*, *L. major*, *L. amazonensis*, and *L. braziliensis*, a robust TRAP result is only achieved with extension primers ending in TTAGGG (Figure S3). This fact might explain the low activity and processivity when using Kim’s TRAP original extension primer [16], suggesting that, once synthesized, the telomeric end of *Leishmania* is further processed. This phenomenon does not occur in *T. cruzi* or *T. brucei*; its significance is unknown.

Considering *L. major* telomerase activation and thermostability, during its life cycle, the highest temperature the parasite experiences is that of its mammalian host (~37 °C), as shown by De Oliveira et al. (2021) [38] in *L. amazonensis*; in the transition from metacyclic promastigotes to amastigotes, the telomerase activity increases when the culture is incubated at 37 °C. In our hands, *Leishmania* promastigote cells divide every 18 h at 25 °C; therefore, we assume that this parasite has a very active telomerase. The higher activity and processivity of *L. major* telomerase beyond physiological temperatures is a property shared by thermostable retrovirus reverse transcriptases such as HIV, Avian myeloblastosis virus (AMV), and Murine Moloney leukemia (M-MLV) [39,40], and it is likely an ancient feature of type II introns, from which telomerase is believed to be derived [41,42,43,44]. Like viral reverse transcriptases, an increase in the temperature unleashes the retrotranscription activity in *L. major* telomerase. Furthermore, our structural homology search and the structural alignment analysis revealed that the *L. major* TERT catalytic site has significant structural homology with the thermostable group II intron reverse transcriptase III from *Geobacillus stearothermophilus* [23] (Figure 6).

Perhaps, heating reveals the structural plasticity of *L. major* TERT, which allows this enzyme to experience conformational changes to adapt to the changing environments of its cell cycle. *In vivo*, these conformational changes might be caused by variations in temperature and the action of specific activators. In humans, Sayed et al. 2019 [45] detected an intracellular telomerase-activator factor (iTAF), which reactivates the enzyme after one elongation step, and in *L. amazonensis*, de Oliveira et al. [38] reported HSP90 as an activator-modulator of telomerase. In this regard, the *Leishmania* TERT is larger than other TERT molecules and contains extensive loops with no homology with other known telomerases (Figure S4); this feature gives room for multiple interactions with other molecules like HSP90 [38].

In our SDS-PAGE experiments, we consistently observed a slight change in the mobility of the TERT sub-unit after heating the nuclear extracts or the immunopurified enzyme. We have no explanations for this mobility behavior; however, since we did not expose the immunoprecipitated enzyme to harsh denaturing treatments except by mixing the samples with a loading buffer containing SDS that detached the TER sub-unit, we attributed these results to reversible conformational changes that affected the number of SDS molecules bound to the protein [46,47,48] (RESULTS, *Structural Simulations*).

Finally, we explored whether *L. major* can survive telomerase inhibition by activating alternative telomeric elongation mechanisms. To this aim, we first used the human telomerase inhibitors MST-312 and TMPyP4 and tested their effect on the activity of the immunopurified enzyme. These inhibitors are effective against human telomerase without affecting the Taq polymerase of the TRAP assay. TMPyP4 is a cationic porphyrin that stabilizes quadruplex formation, thus preventing telomerase extension [24]. Since MST-312 was more potent than TMPyP4 *in vitro*, we selected it to test the effect of telomerase inhibitors on *Leishmania* promastigote cell growth. With inhibitor concentrations ranging from 10 nM to 100 µM (Figure 8), we obtained a cell growth arrest of 70% with 20 nM when we doubled the inhibitor concentration in the fourth week. We did not perform assays with human cancer cells, but other authors [25] reported that a fifty-fold higher concentration was necessary to reach the same cell growth arrest with this inhibitor (1 µM). However, in general, a rapid effect of telomerase inhibitors on cell growth is not expected because it depends on the length of the telomeres. These results emphasize that telomerase is an important activity for proliferating *L. major* forms and that assay conditions ought to be optimized for its proper determination. Lastly, although we have shown that typical telomerase inhibitors abrogate *L. major* telomerase activity, we still cannot rule out the possibility that when exposed for a long time to inhibitors, *Leishmania* can activate alternative mechanisms [9] for telomeric recovery (see the slippage model in Figure S1). In this sense, Bussotti et al. (2018) [49] demonstrated dynamic genomic changes among *Leishmania* species, including non-orthodox telomeric amplification processes. More recently, Oliveira et al. (2024) [50] reported that a double knock-out of the presumptive *L. major* TER sub-unit impacted the parasite’s fitness, including the shortening of telomeres and overproduction of TERRA RNA; however, mutant cells divided and differentiated into metacyclic promastigotes. Interestingly, despite being a *tour de force*, the work did not show how TER deletion affected the telomerase activity.

Our results with inhibitor MST-312 confirmed that *Leishmania major* cell growth can be arrested over relatively long expositions; perhaps, a combination of telomerase inhibitors with human-repurposed drugs [51] could help in the treatment of leishmaniasis.

## 5. Conclusions

Here, we confirmed that *L. major* has the potential to trigger potent telomerase activity, which is tightly regulated by unknown factors. The chemoresistance and activation properties of *L. major* telomerase may be due to an ancestral property of Intron II elements from where this enzyme is thought to be derived, and which is shared by many viral reverse transcriptases. Additionally, our experiments with telomerase inhibitors suggest that this activity is essential for the parasite’s survival, opening the possibility of being used as a target for chemotherapy treatment. However, considering the genomic plasticity of *Leishmania*, an effective treatment can be only obtained through a combination of drugs.

## Data Availability

The original contributions presented in this study are included in the article/Supplementary Material. Further inquiries can be directed to the corresponding author.

## Supplementary Materials

The following supporting information can be downloaded at: https://zenodo.org/uploads/14543863, uploaded on 22 December 2024. Reference [52] are cited in the Supplementary Materials.
